# Machine learning with multimodal neuroimaging data to classify stages of Alzheimer’s disease: a systematic review and meta-analysis

**DOI:** 10.1007/s11571-023-09993-5

**Published:** 2023-08-18

**Authors:** Modupe Odusami, Rytis Maskeliūnas, Robertas Damaševičius, Sanjay Misra

**Affiliations:** 1 Department of Multimedia Engineering, Kaunas University of Technology, Kaunas, Lithuania; 2 Faculty of Applied Mathematics, Silesian University of Technology, Gliwice, Poland; 3 Department of Applied Data Science, Institute for Energy Technology, Halden, Norway

**Keywords:** Alzheimer’s disease stages, Machine learning, Multimodal neuroimaging, Meta-analysis, Systematic review

## Abstract

In recent years, Alzheimer’s disease (AD) has been a serious threat to human health. Researchers and clinicians alike encounter a significant obstacle when trying to accurately identify and classify AD stages. Several studies have shown that multimodal neuroimaging input can assist in providing valuable insights into the structural and functional changes in the brain related to AD. Machine learning (ML) algorithms can accurately categorize AD phases by identifying patterns and linkages in multimodal neuroimaging data using powerful computational methods. This study aims to assess the contribution of ML methods to the accurate classification of the stages of AD using multimodal neuroimaging data. A systematic search is carried out in IEEE Xplore, Science Direct/Elsevier, ACM DigitalLibrary, and PubMed databases with forward snowballing performed on Google Scholar. The quantitative analysis used 47 studies. The explainable analysis was performed on the classification algorithm and fusion methods used in the selected studies. The pooled sensitivity and specificity, including diagnostic efficiency, were evaluated by conducting a meta-analysis based on a bivariate model with the hierarchical summary receiver operating characteristics (ROC) curve of multimodal neuroimaging data and ML methods in the classification of AD stages. Wilcoxon signed-rank test is further used to statistically compare the accuracy scores of the existing models. With a 95% confidence interval of 78.87–87.71%, the combined sensitivity for separating participants with mild cognitive impairment (MCI) from healthy control (NC) participants was 83.77%; for separating participants with AD from NC, it was 94.60% (90.76%, 96.89%); for separating participants with progressive MCI (pMCI) from stable MCI (sMCI), it was 80.41% (74.73%, 85.06%). With a 95% confidence interval (78.87%, 87.71%), the Pooled sensitivity for distinguishing mild cognitive impairment (MCI) from healthy control (NC) participants was 83.77%, with a 95% confidence interval (90.76%, 96.89%), the Pooled sensitivity for distinguishing AD from NC was 94.60%, likewise (MCI) from healthy control (NC) participants was 83.77% progressive MCI (pMCI) from stable MCI (sMCI) was 80.41% (74.73%, 85.06%), and early MCI (EMCI) from NC was 86.63% (82.43%, 89.95%). Pooled specificity for differentiating MCI from NC was 79.16% (70.97%, 87.71%), AD from NC was 93.49% (91.60%, 94.90%), pMCI from sMCI was 81.44% (76.32%, 85.66%), and EMCI from NC was 85.68% (81.62%, 88.96%). The Wilcoxon signed rank test showed a low P-value across all the classification tasks. Multimodal neuroimaging data with ML is a promising future in classifying the stages of AD but more research is required to increase the validity of its application in clinical practice.

## Introduction

AD is among the most widespread neurological conditions, affecting over 20 million people globally, and is expected to rise further in the next decades (Stefano et al. 2019). It typically starts in middle or old age. AD is typically considered an irreversible disease without a cure. Both the cardinal clinical symptoms and the disease proteins can be used to classify neurodegenerative diseases, and AD is classified as tauopathies based on the protein (Jeromin and Bowser 2017). The abnormal accumulation of tau protein and amyloid beta (Aβ) caused tauopathies. Although, the pathophysiologic knowledge of Alzheimer’s disease derived from existing ideas such as amyloid beta deposition has greatly aided understanding of the disease process. Amyloid βeta may begin to build up in the brain 20 years before the first indication of AD occurs, whereas the accumulation of Tau protein occurs 15 years before the first symptoms of AD appear (Goenka and Tiwari 2021). The use of biomarkers that indicate pathophysiological alterations suggesting the development of AD has contributed significantly to the effort to identify the disease as early as feasible. Researchers are identifying and refining radiological which is not limited to neuroimaging, genetic, CSF multisensory, speech, electroencephalogram (EEG), and blood biomarkers. Simultaneously, clinical trials are evaluating the impact of biomarkers that potentially slow or stop the progression of AD. Some of the neuroimaging modalities such as functional magnetic resonance imaging (fMRI), fluorodeoxyglucose positron emission tomography (FDG-PET), structural Magnetic resonance imaging (sMRI), and Diffusion Tensor imaging (DTI) have revealed the related structural and behavioral alterations in the brain during the illness process.

The pathogenic aspect of AD was shown by sMRI scanning, which is frequently used to assess morphometric alterations in the brain associated with the loss of synapses, neurons, and dendritic de-arborization in AD progression over time (Salvatore et al. 2018; Dubois et al. 2021). However, structural imaging is insufficient to reflect changes preceding protein buildup. Analysis has shown that metabolic alterations occur before atrophy in people at risk for AD and a functional biomarker can be identified before the specific protein profiles connected to advanced AD using FDG-PET (Ou et al. 2019; Veitch et al. 2022; Kim et al. 2022). Considering the progression of AD and cognitive impairment, fMRI techniques can track AD-related brain damage (Hojjati et al. 2017; Ahmadi et al. 2021; Li et al. 2020). DTI gives information on the structure of the brain in the form of Mean Diffusivity (MD), Fractional Anisotropy (FA), and Echo Planar Imaging (EPI) intensities (De and Chowdhury 2021). Additionally, DTI can spot early microstructural changes in AD patients before they manifest as gross anatomical changes, changes that standard MRI typically misses.

Mild but measurable changes in thinking ability are seen in people with Mild Cognitive Impairment (MCI), and MCI patients have a high chance of developing AD (Kang et al. 2020). MCI is a medical disorder with symptoms that differ from those associated with normal aging. Depending on different stages, Progressive mild cognitive impairment (pMCI), stable mild cognitive impairment (sMCI) (Lu et al. 2022) early mild cognitive impairment (EMCI), and late mild cognitive impairment (LMCI) (Rallabandi et al. 2020), are the four categories under which MCI can be classified. A fundamental change in the assessment of biomarkers / cognitive markers to predict the transition from MCI to Alzheimer’s disease is needed.

Deep Learning (DL) approaches have been used to handle AD diagnosis difficulties successfully in recent years by applying them to neuroimaging single modality. Despite efforts to diagnose AD in the early stages with a single modality, the correctness, and dependability of the findings are open to doubt while thinking about the establishment of established standards for precise AD staging along with fewer AD-related physiological markers (Kim et al. 2022). The functional alterations that occur in the brain areas cannot be evaluated by sMRI, and sMRI is inappropriate for capturing alterations before protein synthesis. Although FDG-PET can deliver a more thorough diagnosis of brain metabolic cognitive function but might not be appropriate to identify the early indications of AD before the neuronal loss occurs.

Considering this, efforts to find a biomarker specific to AD using multimodal neuroimaging data to improve the diagnostic performance of a computer-aided diagnostic (CAD) system have been actively ongoing. The regional distribution of white matter hypometabolism (WMH) associated with Aβ burden, glucose hypometabolism, and gray matter volume reduction has also been examined from MRI and PET (Gaubert et al. 2021; Pham et al. 2022). The pairwise similarity measures for multiple modalities such as VBM-MRI or FDG-PET were utilized for AD analysis (Hao et al. 2020). Furthermore, multimodal connections between tau deposition, gray matter atrophy, hypometabolism, and white matter tract declension in atypical AD were investigated from MRI, PET, and DT (Sintini et al. 2018). The selection of complementary features from each modality is a predominant challenge faced by research communities working in multimodal neuroimaging (Sharma and Mandal 2023). Neuroimaging studies of AD identify different brain regions depending on the imaging modality, and several studies of specific symptoms within AD have been highly inconsistent (Banning et al. 2019). Additionally, the heterogeneity of neuroimaging modality has raised the concern of reproducibility crisis of AD analysis with multimodal neuroimaging data owing to this, a subfield within artificial intelligence (AI), ML, is becoming more common in developing the automatic sophisticated model for multimodal data in early detection of AD.

Earlier this decade, many researchers focused on multimodal learning to gather and combine latent representation data from several neuroimaging techniques. A growing number of studies have looked at MRI and PET extract to learn multilevel and multimodal features by transforming the regional brain images into higher-level characteristics that are more compact (Sarraf and Tofighi 1603; Lu et al. 2018; Abdelaziz et al. 2021; Jin et al. 2022). Similarly, with the recent establishment of multimodal fusion, a growing number of studies have proposed image fusion methods for multimodal neuroimaging analysis in AD diagnosis, and their effectiveness is evaluated using machine learning (ML) algorithms as multimodal classifiers (Lazli et al. 2019; Song et al. 2021). The purpose of the fusion is to have a better contrast, fusion quality, and improved model performance (Muzammil et al. 2020). The successful utilization of multimodal image fusion coupled with ML has shown that it improves the diagnosis of AD (Veshki et al. 2022). The motivation for this study is based on the heterogeneity of neuroimaging modalities and the challenge of the selection of complementary features from each modality (Goenka and Tiwari 2022a). The anatomical and functional changes in the brain linked to AD may be better understood thanks to neuroimaging techniques. However, it is still unclear whether single-modality neuroimaging approaches can reliably and accurately diagnose AD.

This study sought to determine whether multimodal neuroimaging fusion coupled with ML is reliable and effective to distinguish individuals with early symptoms of AD from the terminal stage of AD using a systematic review and measure the effectiveness of its classification using a random effect meta-analysis. A comparable meta-analysis was found in the literature search (Sharma and Mandal 2023), but the procedure utilized in this system is based on (Aggarwal et al. 2021) and is addressing the following Research Questions (RQNs):

- RQN1: What are the main discoveries and methods used to detect AD using multimodal neuroimaging and ML?".
- RQN2: What are the various fusion techniques utilized in multimodal neuroimaging studies to facilitate classification?
- RQN3: What is the percentage usage of various fusion techniques?
- RQN4: What is the diagnostic accuracy of differentiating between various stages of AD?
- RQN5: What are the significant differences in the performance of multimodal neuroimaging fusion for the classification tasks?

The contributions of this study are as follows:

- This study provides a systematic review and meta-analysis of the contribution of machine learning (ML) to the accurate classification of the stages of Alzheimer’s Disease (AD) using multimodal neuroimaging data.
- The study identifies the potential of multimodal neuroimaging data with ML in accurately classifying different stages of AD. The authors conducted an explainable analysis of the classification algorithms and fusion methods used in the selected studies, which can help researchers and practitioners to understand the strengths and limitations of different methods.
- The study provides pooled estimates of sensitivity and specificity for differentiating between AD and healthy control participants, as well as for differentiating between different stages of Mild Cognitive Impairment (MCI) and early MCI from NC. These estimates can help researchers and practitioners to evaluate the performance of different methods and to compare their results.
- The study highlights the need for additional research to increase the validity of the application of multimodal neuroimaging data with ML in clinical practice. This can guide future research and development in this field.

## Methodology

This section explains the study’s research techniques, including the research questions, the search procedure, the criteria for inclusion and exclusion, and the selection execution. The Preferred Reporting Items for Systematic Reviews and Meta-Analyses (PRISMA) report (Moher et al. 2009), was used to conduct and report this systematic review. A systematic review was conducted to locate studies that used multimodal neuroimaging learning or multimodal neuroimaging fusion to categorize AD phases. Only articles published as a full -text English Language articles between January 2016 and August 2022 (included) were chosen. Articles from before 2016 were excluded because of the methodological (deep learning algorithm and multimodal techniques) gap among earlier research and the criteria used to make them hardly comparable.

We carried out a state-of-the-art search adding phrases together using Boolean operators in IEEE Xplore, Science Direct/Elsevier, ACM Digital Library, and PubMed databases. The relevant subject search terms used are Term A: “Multimodal imaging Fusion” OR “Multimodal Learning”, Term B: “Alzheimer Disease”, Term C: “Mild Cognitive Impairment” OR “MCI”, Term D: “deep learning”. Forward snowballing was also performed on Google Scholar to find any relevant articles. The following rule was created by combining these search keywords: Term A AND Term B AND Term C AND Term D. The eligibility criteria were applied after the removal of duplicates to only choose the articles that included (1) classification of MCI (EMCI or LMCI or pMCI, sMCI) to AD, Stages of AD was diagnosed using internationally accepted scores (3) use of multimodal neuroimaging data (4) Imaging fusion techniques, a (5) classification techniques utilizing ML algorithms (6) accuracy, sensitivity, and specificity for quantitative analysis.

After choosing the appropriate number of studies, the specified facts were extracted for each study: (1) authors and year of publication, (2) Stages, (3) imaging fusion techniques, (4) classification methods, (5) Validation methods, (6) performance metrics score.

We also carried out an explainable analysis based on the systematic evaluation conducted on the commonly used XAI algorithms (Jin et al. 2020). These authors focused on Post-hoc XAI algorithms in their evaluation which explained trained black-box models by probing model parameters and categorized Post-hoc XAI into three: Activation-based, Gradient-based, and Perturbation-based. We further classified the fusion methods into abstraction levels and Performance evaluation analysis of image fusion algorithms based on evaluation conducted by Hermessi et al. (2021).

Data synthesis and analysis were carried out using a metadta statistical program that pools diagnostic test data in Stata. The HSROC model is applied to calculate pooled sensitivity and specificity of selected studies. within- and between-study heterogeneity, along with the correlation between sensitivity and specificity, are all taken into consideration by the hierarchical model (Lee et al. 2015). The command "metandi tp fp fn tn" is used to get the diagnostic odds ratio (DOR), pooled sensitivity, pooled specificity, and likelihood ratio (LR). HSROC is achieved by utilizing the command command “*metandiplot tp fp fn tn”.* Studies with the same type of diagnosis are considered for meta-analysis. Wilcoxon signed-rank test (Derrac et al. 2011), is utilized to statistically compare the accuracy scores of the existing models and determine if there are significant differences in their performance when using multimodal neuroimaging fusion for the classification of pMCI versus sMCI, MCI versus NC, AD versus NC, and EMCI versus NC.

## Results

### Search and Study selection

**Fig. 1:**
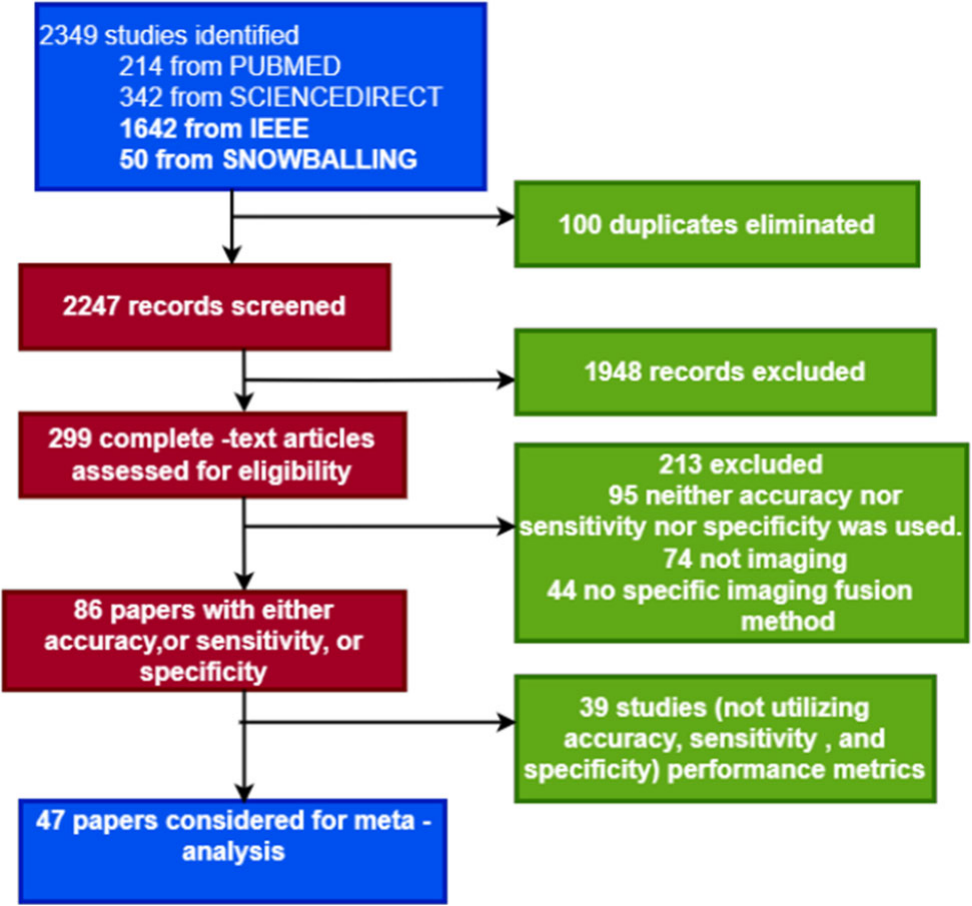
Flow Diagram of Selected Studies using PRISMA Chart

The flow of the survey procedure, as shown in Fig. 1, depicts the analytical review process and the selection of relevant articles at various phases. Database search yielded 2299 results, forward snowballing (Google Scholar) yielded another 50 records, and a total number of 2349 studies were returned from the search. After removing duplicates found due to the combined search, 2247 abstracts were screened. Of these, the 1948 articles did not fulfill the eligibility criteria based on title, abstract, and conclusion. Two hundred and ninety-nine full papers were individually accessed, and 213 Papers were excluded at this stage. 47 papers fulfilled inclusion criteria for the systematic review and contained data for accuracy, sensitivity, and sensitivity for meta-analysis as depicted in Fig. 1, and this expressed the generic answer to RQN1 while the details are provided in subsections of this section.

Summary of the selected studies are represented in Table 1 while Table 2 provides the attributes of the included participants.

**Table 1:** Summary of Multimodal Neuroimaging Fusion Methods in Alzheimer’s Disease Classification

Authors	Year	Imaging modality	Fusion method	ML model	Diagnosis	Internal validation	External validation	Sen (%)	Spe (%)	Acc (%)
Suk et al. (2016)	2016	MRI and PET + Genetics	Sparse multi-task learning	Deep weighed	AD vs NC MCI vs NC	tenfold cross-validation	No	92.0 90.7	98.0 56.0	95.0 78.7
Zheng et al. (2016)	2016	sMRI and PET	Stacked deep polynomial network	Support vector machine (SVM)	AD vs CN	tenfold cross-validation	No	96.8	97.5	97.3
Lei et al. (2016)	2016	PET, sMRI	Multi kernel learning	SVM	AD vs NC NC vs MCI MCIc vs MCInc	tenfold	No	97.8 95.1 77.3	93.6 52.7 95.1	96.9 86.5 82.8
Schouten et al. (2016)	2016	fMRI, sMRI, DTI	Stepwise concatenation	elastic net classifier	Mild AD vs CN Moderate AD vs CN	10- fold	No	72.1 81.3	93.5 95.6	89.6 93.0
Tong et al. (2016)	2017	sMRI, PET + Genetics	The nonlinear graph fusion	Random Forest	AD vs NC MCI vs NC AD vs. MCI vs. NC	Train/test split 75%,25%	No	88.9 85.1 -	94.7 67.1 -	91.8 79.5 60.2
Ahmed et al. (2017)	2017	sMRI, DTI	Multi kernel learning	SVM	AD vs CN CN vs MCI AD vs MCI	10- fold	No	82.9 71.6 65.6	97.2 86.1 81.3	90.2 79.4 76.6
Mathotaarachchi et al. (2017)	2017	PET, demographic, and genetics	Logistic regression analysis	Random Forest	sMCI vs pMCI	tenfold	Yes	71.0	87.0	84.0
Cheng and Liu (2017)	2017	sMRI and PET	High-level cascaded CNN	2D- CNN	AD vs CN	tenfold cross-validation	No	87.1	92.0	89.6
Qiu et al. (2018)	2018	MRI, MMSE, logical memory	locally weighted clustering coefficients	Graph Transformer Network	NC vs. EMCI EMCI vs. LMCI	fivefold	No	92.0 87.0	97.6 85.8	87.0 87.4
Forouzannezhad et al. (2018)	2018	MRI and PET and neurological test scores	Principal component analysis	Deep neural network	NC vs EMCI NC vs LMCI EMCI vs LMCI EMCI vs AD	tenfold	No	83.2 80.4 80.6 87.7	84.4 87.6 60.5 92.2	84.0 84.1 69.5 90.3
Shi et al. (2018)	2018	sMRI, PET	Basic Polynomial Network	SVM	MCI vs CN MCIc vs MCInc AD vs CN	tenfold	No	97.9 68.0 95.9	67.0 86.8 98.5	87.2 78.9 97.1
Liu et al. (2018)	2018	sMRI, PET	3D-CNN	2D-CNN	pMCI vs. CN AD vs. CN sMCI vs. CN	tenfold	No	81.0 92.5 63.0	84.3 93.9 67.3	82.9 93.2 64.0
Kim and LeeLee (2018)	2018	MRI, FDG-PET, CSF measurement	sparse hierarchical extreme learning machine	SVM	AD vs. HC MCI vs. HC	tenfold	No	97.0 75.1	91.1 91.9	96.0 86.1
Khvostikov et al. (2018)	2018	sMRI, DTI	CNN Autoencoder	3D- CNN	MCI vs CN AD vs MCI AD vs CN	Train/Test in 90%/10% partition	No	85.8 93.3 95.8	62.4 99.2 97.5	62.5 80.0 96.7
Aderghal et al. (2018)	2018	sMRI, DTI	CNN	CNN	AD vs. CN AD vs. MCI MCI vs. CN	Random split	No	94.7 93.7 92.8	90.4 79.1 73.0	92.5 85.0 80.0
Ortiz et al. (2018)	2018	sMRI, PET	K-singular value representation	SVM	AD vs CN MCI vs CN	10 -fold	No	94.0 85.0	89.0 71.0	92.0 79.0
Choi and Jin (2018)	2018	FDG-PET, AV45 PET	Deep learning	Deep CNN	AD vs CN MCInc vs MCIc	tenfold	No	93.6 81.0	97.8 87.0	96.0 84.2
Lu et al. (2018)	2018	sMRI, PET	Deep neural network	Deep neural network	sMCI vs. pMCI	tenfold	No	79.7	83.8	82.9
Feng et al. (2018)	2018	sMRI, PET	SBi-RNN	SVM	AD vs. NC pMCI vs. NC sMCI vs. NC	tenfold	No	96.6 83.4 70.4	92.4 85.4 48.4	94.3 84.7 64.5
Peng et al. (2019)	2019	sMRI, PET, and genetic data	Multiple kernel learning	SVM	AD vs NC MCI vs NC AD vs MCI	tenfold	No	97.3 79.8 65.9	94.9 85.6 82.7	96.1 80.3 76.9
Lee et al. (2019)	2019	MRI, CSF, Cognitive measures, Demographic	Different RNN-GRU on each modality and to be concatenated	Linear regression classifier	MCIc vs MCInc	5- fold	No	83.0	77.0	79.0
Zhang et al. (2019)	2019	MRI and PET, cognitive measures	the weighted average of correlation analysis	3DCNN	AD vs MCI MCI vs NC AD vs NC	Train/test split 90% to 10%	No	97.4 90.1 96.5	84.3 91.8 95.3	88.2 85.7 98.4
Ezzati et al. (2019)	2019	MRI, APOE4 genotype, demographic measures	Multitask learning	Ensemble linear discriminant	AD vs CN	Random split	No	95.8	88.3	92.8
Feng et al. (2019)	2019	sMRI, PET	FSBI-RNN	RNN	AD vs. CN pMCI vs. CN sMCI vs. CN	tenfold		97.7 83.3 70.6	92.4 88.8 59.6	94.8 86.4 65.4
Huang et al. (2019)	2019	sMRI, PET	Multitask learning (CNN)	VGG-16	CN vs. AD sMCI vs. pMCI	Train/Test split in 70% to 30%	No	90.9 73.4	89.2 71.2	90.1 72.2
Hojjati et al. (2019)	2019	sMRI,fMRI	discriminant correlation analysis (DCA)	SVM	MCIc vs MCInc MCIc vs MCInc vs NC AD vs MCIc vs MCInc vs NC	fivefold	No	59.3 61.7 51.8	78.7 79.3 79.9	59 61.6 52
Xing et al. (2019)	2019	sMRI, fMRI	dynamic spectral graph convolution networks	LSTM	CN vs EMCI	Random split	No	86.5	72.9	79.7
Kang et al. (2020)	2020	sMRI, DTI	VGG16	SVM	EMCI vs CN	fivefold	No	97.3	92.9	94.2
Hao et al. (2020)	2020	sMRI, PET	Consistent metric	SVM	AD vs CN LMCI vs CN EMCI vs LMCI	tenfold	No	98.4 94.0 67.4	96.7 66.1 85.5	93.7 78.5 73.9
Gupta et al. (2020)	2020	sMRI, FDG, AV45, DTI, rs-fMRI, and APOE	Multiple kernel learning	SVM	AD vs. NC MCInc vs. MCIc NC vs MCIc	Train/Test in 70% / 30% partition	No	94.1 100 100	100 90.9 88.8	96.0 94.7 94.2
Shao et al. (2020)	2020	sMRI, PET	Hypergraph Laplacian matrix	SVM	AD vs. CN LMCI vs. CN EMCI vs. LMCI	tenfold	No	94.1 86.1 83.8	90.4 78.6 63.3	92.5 82.5 75.5
Zhang and Shi (2020)	2020	sMRI, PET	Attention mechanism	ResNet	AD vs MCI sMCI vs pMCI	Train/Test in 60% / 40% partition	No	93.4 81.2	97.5 93.5	95.2 89.8
Lei et al. (2020)	2020	fMRI and DTI	Self-calibrated indicators	SVM	NC vs EMCI NC vs LMCI EMCI vs LMCI	Leave -one-out	No	86.4 90.9 78.95	84.1 84.2 84.1	85.2 87.8 81.71
Yu et al. (2020)	2020	fMRI and DTI	Sparse graph construction	CNN	EMCI vs. NC LMCI vs. NC LMCI vs. EMCI	leave-one-out	No	86.5 94.0 93.5	84.4 92.5 90.0	85.4 93.4 92.3
Fang et al. (2020)	2020	sMRI and PET	CNN	Adaboost	AD vs MCI EMCI vs NC	Train /Test split in 65% to 35%	No	89.7 88.3	93.5 92.5	92.5 90.3
Pan et al. (2021a)	2021	fMRI,DTI	Sparse Graph	SVM	CN vs. SMC CN vs EMCI CN vs LMCI	Leave one out cross-validation	No	88.6 86.4 84.2	77.2 84.1 90.9	82.9 85.2 87.8
Wang et al. (2021)	2021	sMRI, fMRI	kernel canonical correlation analysis	SVM	CN vs AD AD vs MCI CN vs MCI LMCI vs EMCI	Train/ Test split in the ratio 70% to 30%	No	100 100 100 100	93.3 100 100 100	96.0 100 100 100
Zuo et al. (2021)	2021	sMRI, fMRI,DTI	adversarial hypergraph fusion	CNN	AD vs. NC LMCI vs. NC EMCI vs. NC	tenfold	No	93.7 94.7 86.7	96.1 88.9 88.5	95.1 91.5 87.5
Ning et al. (2021)	2021	sMRI,PET	Relational regularization feature selection	2DCNN	AD vs. NC pMCI vs. sMCI MCI vs. NC	tenfold	No	95.6 94.8 91.0	97.8 90.9 65.7	96.9 84.5 82.6
Zhang et al. (2021)	2021	sMRI,PET	3D residual attention	3DCNN	AD vs CN	tenfold	No	83.6	93.4	90.3
Lao and Zhang (2022)	2022	sMRI, fMRI	3D discrete wavelet transforms	Stacked autoencoders	AD vs CN pMCI vs sMCI CN vs MCI AD vs MCI	fivefold	No	93.5 89.7 78.4 78.6	100 83.5 75.1 88.1	96.9 87.7 76.6 84.2
Meng et al. (2022)	2022	fMRI, DTI	Multi-modal LassoNet	Shallow neural network	AD vs CN AD vs EMCI CN vs EMCI	Train/ Test split in the ration 60% to 40%	No	88.8 87.2 90.9	91.9 79.0 87.1	90.7 83.6 88.8
Xu et al. (2022)	2022	sMRI, PET	three sine–cosine fusion (SCF)	CNN	MCI vs CN sMCI vs pMCI	Random split	No	82.1 84.1	76.7 85.6	80.2 84.9
Dwivedi et al. (2022)	2022	sMRI, PET	discrete wavelets transform	SVM	CN vs AD CN vs MCI AD vs MCI	tenfold	No	97.0 97.0 96.0	97.0 91.0 99.0	97.0 94.0 97.5
Jia and Lao (2022)	2022	sMRI, fMRI	Kernel canonical correlation analysis	SVM	NC vs. SMC NC vs. MCI SMC vs. MCI SMC vs. AD MCI vs. AD NC vs. AD	Train/Test split 70% to 30%	No	100 93.3 100 87.5 90.0 100	75.0 90.0 87.5 100 100 80.0	91.3 92.0 94.4 94.4 95.0 92.0
Pan and wang (2022)	2022	fMRI, DTI	Attention mechanism	Vision Transformer	AD vs. NC LMCI vs. NC EMCI vs. NC	Random split	No	93.3 90.0 90.4	95.2 95.2 95.0	94.4 93.5 92.6
Dong et al. (2022)	2022	sMRI, PET	Latent feature representation	SVM	AD vs. NC MCI vs. NC AD vs. MCI	tenfold	No	83.6 71.3 70.3	80.5 66.1 70.3	81.8 71.1 70.8

**Table 2:** Characteristics of Participants in the Included Study

Study	Size	Source
*MCI/NC*		
Dong et al. (2022)	52/52	ADNI
Jia and Lao (2022)	35/50	ADNI
Lao and Zhang (2022)	203/181	ADNI
Ning et al. (2021)	200/99	ADNI
Peng et al. (2019)	125/100	ADNI
Ortiz et al. (2018)	111/68	ADNI
Aderghal et al. (2018)	443/241	ADNI
Khvostikov et al. (1801)	108/58	ADNI
kim and lee (2018)	99/52	ADNI
Shi et al. (2018)	99/52	ADNI
Ahmed et al. (2017)	58/52	ADNI
Suk et al. (2016)	99/52	ADNI
Tong et al. (2016)	74/36	ADNI
Lei et al. (2016)	204/101	ADNI
*AD/NC*		
Dwived et al. (2022)	100/100	ADNI
Meng et al. (2022)	23/23	ADNI
Zhang et al. (2021)	282/603	ADNI
Wang et al. (2021)	34/50	ADNI
Shao et al. (2020)	160/160	ADNI
Hao et al. (2020)	160/211	ADNI
Ezzati et al. (2019)	249/424	ADNI
Feng et al. (2019)	93/100	ADNI
Feng et al. (2018)	93/100	ADNI
Cheng and Liu (2017)	93/100	ADNI
Zheng et al. (2016)	51/52	ADNI
Schouten et al. (2016)	77/173	OASIS
*pMCI/sMCI*		
Xu et al. (2022)	55/138	ADNI
Zhang and Shi (2020)	105/13	ADNI
Gupta et al. (2020)	31/30	ADNI
Lee et al. (2019)	163/376	ADNI
Huang et al. (2019)	326/441	ADNI
Hojjati et al. (2017)	25/69	ADNI
Marcus et al. (2007)	702/409	ADNI
Choi and Jin (2018)	79/92	ADNI
Mathotaarachchi et al. (2017)	43/230	ADNI
*LMCI/NC*		
Pan et al. (2021b)	80/84	ADNI
Zou et al. (2021)	82/78	ADNI
Lei et al. (2020)	44/44	ADNI
Fang et al. (2020)	297/251	ADNI
Yu et al. (2020)	39/28	ADNI
Kang et al. (2020)	70/50	ADNI
Pan and Wang (2022)	44/ 44	ADNI
Xing et al. (2019)	191/177	ADNI
Forouzannezhad et al. (2018)	296/248	ADNI
Qiu et al. (2018)	44/44	ADNI

### Datasets

All studies analyzed in this systematic review used the ADNI dataset, except the study (Schouten et al. 2016) that used the OASIS dataset.

The Alzheimer’s Disease Neuroimaging Initiative (ADNI) (Marcus et al. 2007) dataset is a publicly available dataset that has been used in many studies on Alzheimer’s Disease (AD). ADNI is a collaborative research effort involving multiple institutions and funded by the National Institutes of Health. The ADNI dataset includes longitudinal data from individuals with AD, Mild Cognitive Impairment (MCI), and healthy control (NC) participants. The data includes clinical assessments, cognitive tests, genetic information, and multimodal neuroimaging data from Magnetic Resonance Imaging (MRI), Positron Emission Tomography (PET), and cerebrospinal fluid biomarkers. The data typically includes images of brain structures, such as gray matter, white matter, and cerebrospinal fluid, as well as functional images of brain activity, such as regional cerebral blood flow or glucose metabolism.

The Open Access Series of Imaging Studies (OASIS) (Pan et al. 2021b) dataset is a publicly available dataset that contains neuroimaging data and clinical information from individuals with and without dementia. The dataset was created to provide a resource for researchers to study the brain and its changes over time in the context of normal aging and neurodegenerative diseases such as Alzheimer’s disease. The OASIS dataset includes T1-weighted MRI scans, demographic information, and cognitive test scores from over 1,500 individuals. The dataset is divided into two subsets: a cross-sectional dataset and a longitudinal dataset. The cross-sectional dataset includes MRI scans and clinical data from over 400 individuals with Alzheimer’s disease, mild cognitive impairment, and cognitively normal individuals. The longitudinal dataset includes MRI scans and clinical data from over 500 cognitively normal individuals, some of whom went on to develop cognitive impairment or Alzheimer’s disease during the study period. The OASIS dataset has been widely used in research on Alzheimer’s disease and other neurodegenerative diseases, as well as in studies on normal aging and brain development. It has contributed to the development and validation of machine learning models for Alzheimer’s disease diagnosis and classification, as well as to the study of structural changes in the brain over time(Pan et al. 2021b). The OASIS dataset is a valuable resource for researchers studying the brain and its changes over time in the context of aging and neurodegenerative diseases.

The multimodal neuroimaging data used in these datasets provide a rich source of information for machine learning algorithms to identify patterns and classify different stages of AD.

### Baseline methods

Baseline methods for AD recognition and stage classification using the ADNI dataset typically involve using clinical and cognitive assessments, as well as neuroimaging data such as Magnetic Resonance Imaging (MRI) and Positron Emission Tomography (PET) scans. In terms of clinical assessments, commonly used measures include the Mini-Mental State Examination (MMSE), Clinical Dementia Rating (CDR), and the Alzheimer’s Disease Assessment Scale-Cognitive subscale (ADAS-Cog). These assessments can help diagnose and stage AD based on the severity of cognitive impairment. Neuroimaging data can also be used for AD recognition and stage classification. MRI scans can be used to measure brain volume, cortical thickness, and hippocampal atrophy, which are all known to be associated with AD. PET scans can be used to measure the accumulation of beta-amyloid and tau proteins, which are also biomarkers of AD. Baseline methods for AD recognition and stage classification using the ADNI dataset typically involve using these clinical and cognitive assessments, as well as neuroimaging data, to identify individuals with AD or mild cognitive impairment, and to differentiate them from healthy control participants. Machine learning algorithms can be applied to these baseline methods to develop more accurate and objective methods for Alzheimer’s disease recognition and stage classification. Several machine learning algorithms can be used as baseline methods for AD recognition and stage classification using the ADNI dataset. Here are some examples:

- Logistic regression: This is a type of linear model that can be used for binary classification problems (e.g., AD vs. healthy controls). Logistic regression can be used to model the relationship between the input features (e.g., clinical assessments, and neuroimaging data) and the binary outcome variable (e.g., AD vs. healthy controls).
- Random forest: This is an ensemble learning method that can be used for classification problems. Random forest combines multiple decision trees to make a final prediction. Each tree is trained on a random subset of the input features, and the final prediction is based on the majority vote of all the trees.
- Support vector machines (SVM): This is a type of linear model that can be used for binary classification problems. SVM finds a hyperplane that separates the input data into two classes (e.g., AD vs. healthy controls). The hyperplane is chosen to maximize the margin between the two classes.
- Convolutional neural networks (CNN): This is a type of deep learning model that can be used for image analysis tasks, such as MRI or PET scans. CNNs can automatically learn hierarchical representations of the input data and are commonly used for object recognition tasks. In the context of AD recognition, CNNs can be used to identify patterns in neuroimaging data that are indicative of AD or MCI.

These machine-learning algorithms can be used as baseline methods for AD recognition and stage classification and can provide a starting point for developing more accurate and sophisticated models. It is important to note that the choice of algorithm will depend on the specific task and the characteristics of the input data. Metaheuristics approach such as spider monkey optimization algorithm, Cuckoo Search optimization, Bat Inspired Algorithm, Ant Lion Optimization, and Moth Flame Optimization has been hybridized with ML, and utilized in CoVID-19, lung cancer, Retinal artery vein, Chronic Kidney, and diabetes respectively (Kaur et al. 2023). Metaheuristics with ML have also been utilized in the diagnosis of AD in MRI images (Shankar et al. 2019; Chitradevi et al. 2021; Sayed et al. 2017). However, it is important to note that the utilization of metaheuristics in combination with ML techniques has been limited in the context of multimodal neuroimaging.

### Features of neuroimaging data

The ranking of features of neuroimaging data from the ADNI and OASIS datasets that have the greatest impact on the medical diagnosis and stage classification of AD may vary depending on the specific machine learning algorithm and dataset used. However, some studies have identified specific features that are consistently important across multiple studies. For example, in a study by Liu et al. (2015) that used the ADNI dataset, the authors found that the most important features for distinguishing AD from healthy controls were gray matter volume in the medial temporal lobe and the entorhinal cortex. In a similar study by Kung et al. (2021) that also used the ADNI dataset, the authors found that cortical thickness in the entorhinal cortex and the inferior temporal gyrus were the most important features for distinguishing AD from healthy controls. In another study by Gu et al. (2022) that used the ADNI dataset, the authors found that the most important features for distinguishing AD from healthy controls were gray matter volume in the hippocampus, amygdala, and temporal lobe, as well as cortical thickness in the medial temporal lobe.

In terms of stage classification, some studies have found that different features may be important for distinguishing between different stages of AD. For example, in a study by Guo et al. (2020) that used the ADNI dataset, the authors found that different features were important for distinguishing between mild cognitive impairment and AD, as compared to distinguishing between mild cognitive impairment and healthy controls. Specifically, cortical thickness in the medial temporal lobe and the inferior temporal gyrus were the most important features for distinguishing mild cognitive impairment from healthy controls, while gray matter volume in the hippocampus, amygdala, and entorhinal cortex were the most important features for distinguishing mild cognitive impairment from AD. The most important features of neuroimaging data for medical diagnosis and stage classification of AD appear to be gray matter volume and cortical thickness in regions of the brain associated with memory and cognitive function, such as the hippocampus, amygdala, and medial temporal lobe. However, the exact features that are most important may vary depending on the specific machine learning algorithm and dataset used, and additional research is needed to further understand the underlying neural mechanisms of AD and how they can be detected using neuroimaging data.

### Explainable analysis of the selected studies

The visualization of classification results by the ML models is vital, especially in critical fields like healthcare (Chen et al. 2020). Ensuring that the machine learning model can explain decisions, can also strengthen the possibility to know the model fairness, reliability, and robustness of the model. Explain- ability is also important to debug ML models and make informed decisions about how to improve them. The activation-based method is the most frequently used explanation method for interpreting the predictions of CNN by creating a coarse localization map that highlights the critical areas of the image for the prediction outcome (Selvaraju et al. 2017; Jiang et al. 2021). Gradient-based methods gradient-based visualization methods guided backpropagation, backpropagation, and Grad-CAM are gradient-based visualization methods that determine the gradient of the inference about the input image to retrieve the spatial information of the input called saliency map (Selvaraju et al. 2017; Huff et al. 2021). The perturbation-based method produces a series of perturbed images by modifying the input of the model and observing the changes in the output which are expected to indicate which parts of the input are very important (Ivanovs et al. 2021). The explainable analysis conducted on the forty—seven selected studies is depicted in Table 3

**Table 3:** Explainable Analysis for the Selected Forty-Seven studies

Study	Post-hoc XAI algorithms		
	Activation based	Gradient-based	Perturbation based
Suk et al. (2016)	X	X	X
Zheng et al. (2016)	X	X	X
Schouten et al. (2016)	X	X	X
Lei et al. (2016)	X	X	X
Tong et al. (2016)	X	X	X
Cheng and Liu (2017)	X	X	X
Mathotaarachchi et al. (2017)	X	X	X
Ahmed et al. (2017)	X	X	X
Qiu et al. (2018)	X	X	X
Kim and Lee (2018)	✓	X	X
Shi et al. (2018)	X	X	X
Liu et al. (2018)	X	X	X
Khvostikov et al. (2018)	X	X	X
Aderghal et al. (2018)	X	X	X
Forouzannezhad et al. (2018)	X	X	X
Ortiz et al. (2018)	X	X	X
Choi and Jin (2018)	X	X	X
Lu et al. (2018)	X	X	X
Feng et al. (2018)	X	X	X
Feng et al. (2019)	X	X	X
Huang et al. (2019)	X	X	X
Peng et al. (2019	X	X	X
Lee et al. (2019)	X	X	X
Hojjati et al. (2017)	X	X	X
Zhang et al. (2019)	X	X	X
Xing et al. (2019)	X	X	X
Ezzati et al. (2019)	X	X	X
Kang et al. (2020)	✓	X	X
Hao et al. (2020)	X	X	X
Shao et al. (2020)	X	X	X
Zhang and Shi (2020)	X	X	X
Lei et al. (2020)	X	X	X
Yu et al. (2020)	X	X	X
Gupta et al. (2020)	X	X	X
Fang et al. (2020)	X	X	X
Pan et al. (2021a)	X	X	X
Wang et al. (2021)	X	X	X
Zuo et al. (2021)	X	X	X
Ning et al. (2021)	X	X	X
Zhang et al. (2021)	X	X	X
Lao and Zhang (2022)	✓	X	X
Meng et al. (2022)	X	X	X
Xu et al. (2022)	X	X	X
Dwivedi et al. (2022)	X	X	X
Jia and Lao (2022)	X	X	X
Pan and wang 2022	X	X	X
Dong et al. (2022)	X	X	X

*Note: X, not available for the study;* ✓*, available for the study*

### Categorization of image fusion methods into abstraction levels

**Fig. 2:**
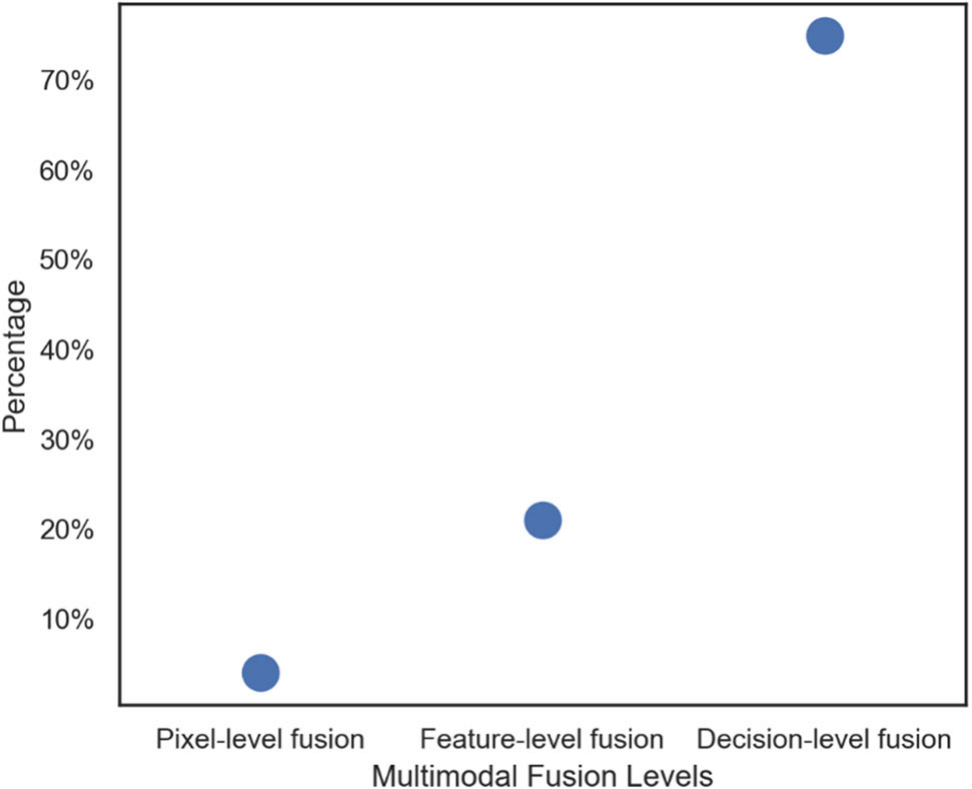
Percentage Usage of Fusion Level based on the Included Studies

**Fig. 3:**
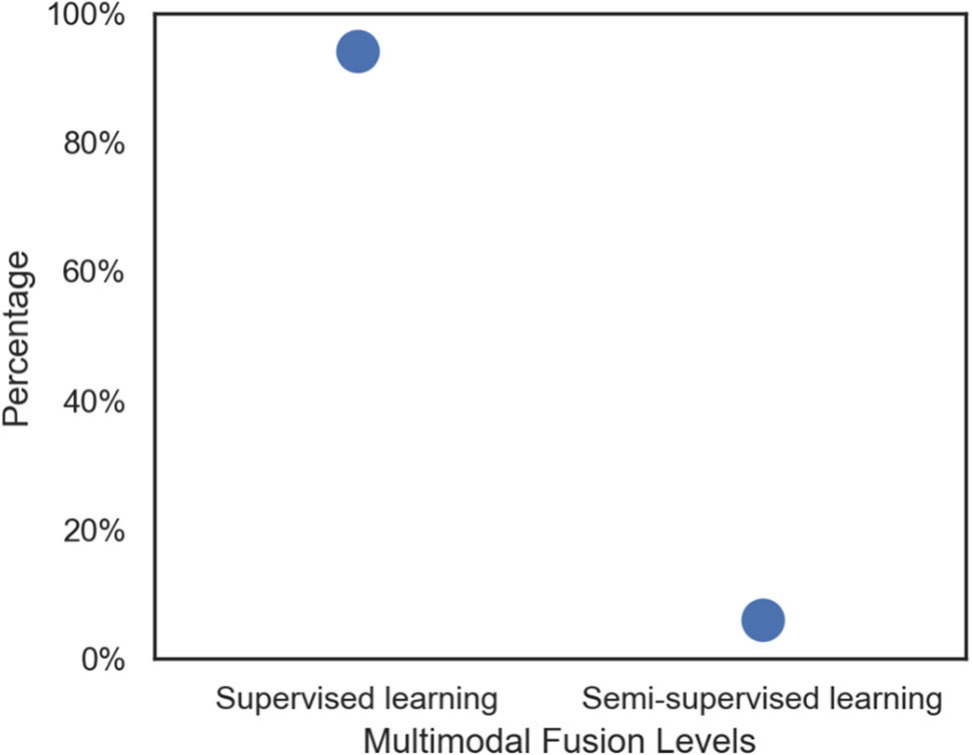
Percentage Usage of Classifiers by the Included Studies

The answers to RQN2 and RQN3 are provided in this section. The goal of image fusion is to create a merged image by combining information from multiple image (Liu et al. 2018) modalities, and the abstraction level at which information is combined when dealing with complementary information needs to be considered. The fusion methods utilized by the forty- seven studies are classified as three abstraction levels: Pixel-level fusion, Feature-level fusion, and Decision-level fusion (Jin et al. 2020). Pixel–level fusion combined multiple input images which could be captured from different imaging devices or a single type under different parameters settings into a fused image (Liu et al. 2018; Liu et al. 2018; Wang et al. 2023). Feature-level image fusion is an intermediate-level fusion based on the comprehensive analysis of feature information extracted from the information of each image source to form fused information(Wang et al. Feb. 2023; Xiao et al. 2020).Decision-level fusion includes fusion at an advanced level and brings together the interpretations of data from different imaging modalities obtained by local decision-makers based on voting, inference, evidence theory, and fuzzy integral (Xiao et al. 2020; Rajini and Roopa 2017). Figures 2 and 3 gives the percentage usage of each of the fusion level and the classifier for the fused information respectively.

Table 4 summarizes the bivariate and HSROC parameter estimates with their standard errors and approximate 95% confidence interval (CI) in Stata. When differentiating MCI from NC, AD from NC, pMCI from sMCI, and EMCI from NC participants the pooled sensitivity was 83.77% with 95% CI (78.87%, 87.71%), 94.60% with 95% CI (90.76%, 96.89%), 80.41% with 95% CI (74.73%, 85.06%), 86.63 with 95% CI (82.43%, 89.95%), while specificity was 79.16% with 95% CI (70.97%, 87.71%), 93.49 with 95% CI (91.60%, 94.90%), 81.44% with 95% CI (76.32%, 85.66%), 85.68% with 95% CI (81.62%, 88.96%), respectively, as depicted in Table 4.

**Table 4:** Summary of Bivariate and HSROC Parameter

No studies/Diagnosis	Parameters	Coefficient	Standard Error	[95% Conf. interval]	
9/pMCI versus sMCI	Sensitivity Specificity	0.80412 0.81443	0.02634 0.02377	0.74733 0.76327	0.85069 0.85661
Covariance between estimates of E(logitSe) and E(logitSp) .0047818					
15 / MCI versus NC	Sensitivity Specificity	0.83775 0.79163	0.02248 0.03709	0.78874 0.70971	0.87716 0.85515
Covariance between estimates of E(logitSe) and E(logitSp) 0 .0011304					
13/AD versus NC	Sensitivity Specificity	0.94602 0.93491	0.01506 0.00846	0.90768 0.91621	0.96898 0.94966
Covariance between estimates of E(logitSe) and E(logitSp) -0.0005566					
10/EMCI versus NC	Sensitivity Specificity	0.86636 0.85684	0.01909 0.01866	0.82431 0.81624	0.89956 0.88968
Covariance between estimates of E(logitSe) and E(logitSp) 0 .013727					

**Fig. 4:**
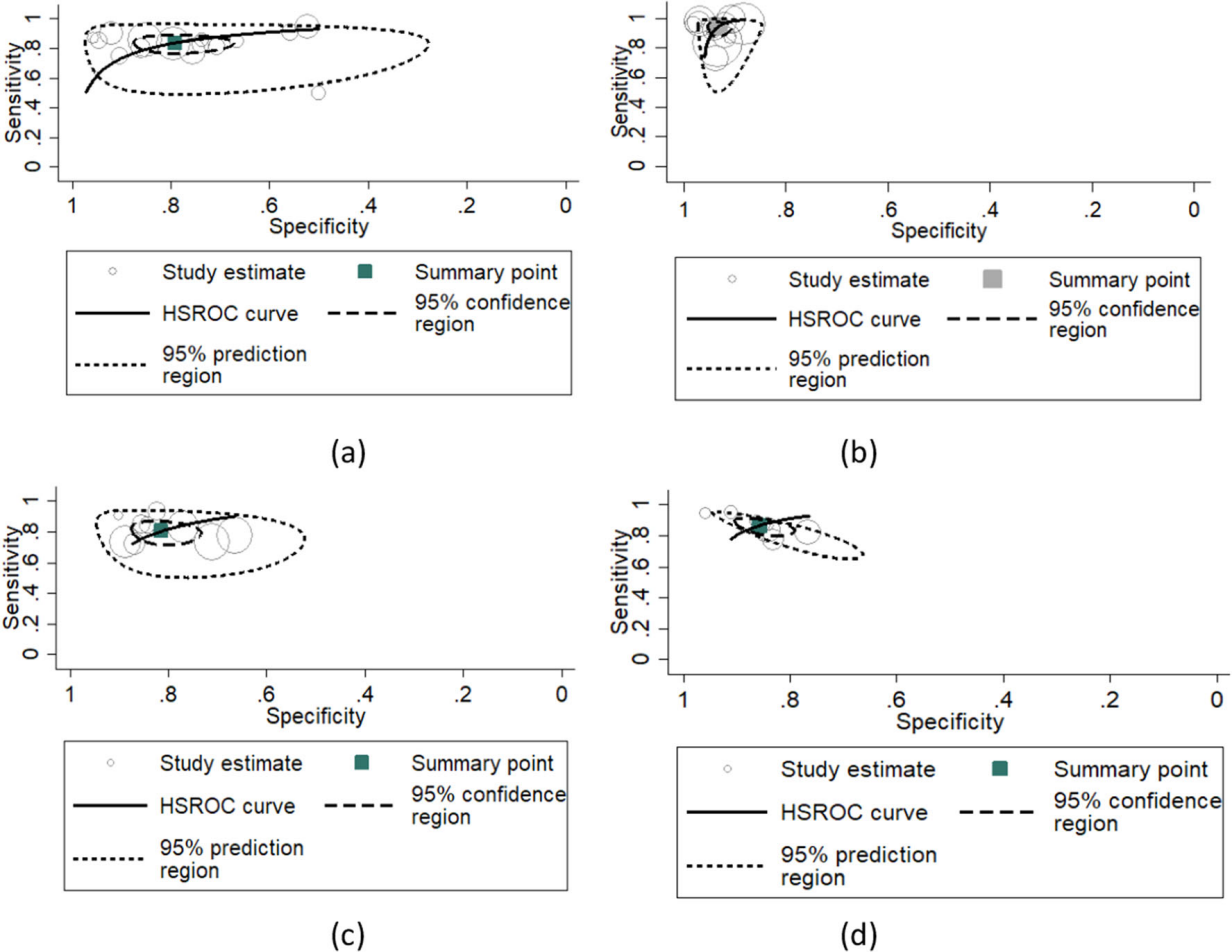
HSROC Curve for Included Studies: **a** MCI vs NC, **b** AD vs NC, **c** pMCI vs sMCI, **d** EMCI vs NC

Figure 4a–d shows the HSROC curve of studies differentiating MCI from NC, AD from NC, pMCI from sMCI, and EMCI from NC participants respectively, and this provides the answer to RQN4. Each study point in Fig. 4 was scaled according to the precision of sensitivity and specificity in the study. The solid circle represents the summary estimate of sensitivity and specificity for each of the diagnosis (MCI vs NC, AD vs NC, pMCI vs sMCI, and EMCI vs NC). The summary point is enclosed by a spotted line denoting the 95% confidence area and a dashed line denoting the 95% prediction area (the area within which one is 95% certain the results of a new study will fall). The pooled DOR for differentiating MCI from NC participants was 19.61% with 95% CI (11.26%, 34.17%), and the pooled DOR for differentiating AD from NC participants was 251.75% with 95% CI (133.30%, 475.44%) while the pooled DOR for differentiating pMCI from sMCI and EMCI from NC participants was 18.01% with 95% CI (11.04%, 29.38%), and 38.80 with 95% CI (22.46%, 67.03%), respectively. Table 5 shows the result of the Wilcoxon signed ranks test for pairwise statistical comparison of the accuracy of the existing model depicted in Table 1 for the classification of pMCI versus sMCI, MCI versus NC, AD versus NC, and EMCI versus NC with 0.90 hypothetical value for comparison. Table 5 provides the answer to RQN5.

**Table 5:** Wilcoxon signed ranks test Result for pairwise statistical

No studies/diagnosis	*P* value
9/pMCI vs sMCI	0.00390
15 / MCI vs NC	0.00006
13/AD vs NC	0.00024
10/EMCI vs NC	0.00195

## Discussion

We looked at research that has already been published on multimodal neuroimaging data with ML algorithms as a fair approach to detecting stages of AD. According to the selected 47 studies in the quantitative analysis, about three studies reported the visualization of feature-level properties using class activation maps. The Post-hoc XAI algorithms for multimodal explanations provided by Jin et al. (2020) are a helpful starting point for the explainable multimodal model. Although most of all the studies included information about the sensitivity and specificity of the model decision the most relevant features to predict AD stages after the fusion of information from different modalities are not analyzed. Only 3 out of the 47 studies presented their results with some visualization of the relevant brain regions for the classification of AD stages. In terms of the fusion methods, we observed that the pixel-level methods (Dwivedi et al. 2022) used techniques based on multiscale decomposition (Wavelet transform), wherein the decomposition transform is used to first break down the source images into multiscale coefficients. Wavelet transforms have been proven to be effective at extracting information details from one image to inject them into another image based on additions, substitutions, or strategy choices. This technique could capture both location and frequency information, and it could extract spatial structures over a range of sizes, thereby being able to separate high frequencies from low frequencies. Most of the studies utilized feature-level methods which operate on features extracted from the images, and the extracted information is achieved using some intelligent computing techniques such as machine learning based methods (Zuo et al. 2021; Xu et al. 2022), region-based algorithms (Pan and Wang 2022), and similarity-matching to content (Dong et al. 2022). Machine learning-based methods (CNN) of multimodality fusion is an effective medical image analysis method (Mathotaarachchi et al. 2017; Huang et al. 2019; Jiang et al. 2021; Liu et al. 2018) for multi-class classification (Goenka and Tiwari 2022b, c). Authors in Daneshtalab et al. (2019) produced an accuracy of 94.2% which is a better performance than that (Qiu et al. 2018) with an accuracy of 84.0%. Both studies fused information extracted from sMRI and DTI images, but the study with machine learning-based methods (Kang et al. 2020) performed better. From Fig. 3, it is shown that feature-level fusion has 75% usage by the included studies.

A preferred abstraction by most of the researchers was feature-level fusion due to its capability of proving more valid results in the case of compatible features (Daneshtalab et al. 2019; Agarwal and Desai 2021). However, the concatenation of compatible features may produce an extremely feature vector that makes the computational load more difficult (Nachappa et al. Apr. 2018). Several other studies used decision-level fusion in which features are ascertained and extracted from each source image, then categorized with regional classifiers, and then decision rules are utilized to combine the information (Peng et al. 2019; Fang et al. 2020). Although decision-level-fusion aimed to support accepted interpretations and comprehensions, the limitation resides in the requirement for prior knowledge-making algorithms to be very complex (Jin et al. 2020; Lahat et al. 2015). Therefore, considering the effect of fusion strategy on the performance of the classification model, we cannot say categorically that a particular fusion strategy is preferable over the others. In all of the fusion levels identified in the included studies, the objective evaluations of the fusion methods’ performances were not considered in all of the included studies, and this evaluation would have helped to assess the image noise, resolution differences between images, and computational complexity from fused images (Kaur et al. 2021). These evaluations would have also provided more insights into the studies utilizing fused images given the percentage of information retained from source images, level of synthetic information produced, and the level of noise (Huang et al. 2020). However, significant progress has been recorded in other domain using pixel fusion-level (Singh et al. 2018; Liu et al. 2021a). Another important finding from this study is that generalization and stability ability of the multimodal model was not further verified as shown in Table 1. None of the studies tested their model in different datasets, and as for validation methods, 29 studies selected cross-validation with different number of folds. Leave -one-out cross validation was selected in 3 studies while random -split validation method was selected in 5 studies. Finally, 10 studies utilized the train/test method of validation.

The results of the meta-analysis are listed in Table 3. We used a bivariate model to directly provide pooled sensitivity and pooled specificity with corresponding 95% CI for four different diagnoses of AD on multimodal neuroimaging data. Sensitivity and specificity are chosen as the main outcome measures in the meta-analysis of diagnostic accuracy studies producing dichotomous index test results because most primary studies report results in pairs of sensitivity and specificity. To the best of our knowledge, this is the first comprehensive review and meta-analysis to look at the diagnostic value of multimodal neuroimaging data for AD diagnosis.

Because the analysis is bivariate, we may test for variations in either sensitivity or specificity or both, between the four diagnoses of AD extracted from the 47 studies. Considering the 47 studies included in the quantitative analysis, the pooled sensitivity and pooled specificity results show that the pooled sensitivity and specificity of studies diagnosing pMCI versus sMCI is significantly lower than that of other studies. It shows that studies with MCI vs NC are a more sensitive test than pMCI versus sMCI, but at the cost of more false positive test findings and a resulting poorer specificity. These results, therefore, suggest favorable sensitivity and specificity of multimodal neuroimaging-based models when compared to single modal neuroimaging–based models. The result of the pooled DOR also indicates heterogeneity between studies, with wide CI indicating the need for more and better-powered studies. The result of the Wilcoxon Signed Ranks Test shows that the obtained P-value from each of the classification tasks is less than the typical significance level of 0.05, which suggests that there is strong evidence to reject the null hypothesis. This indicates that there is a significant difference in the accuracy of the existing models. The results in Table 5 suggest that the classification model can distinguish between individuals with cognitive impairment and those without it, with high accuracy. This finding implies that the classification model is particularly effective at distinguishing between these two groups. The low p-value suggests that the model’s accuracy in classifying individuals as MCI or NC is significantly better than the other three classification tasks.

No study included in the analysis had more than 700 individuals, which raises questions regarding overfitting, most especially for the feature-level fusion. Generally, unsupervised, semi-supervised, supervised, and reinforcement learning are the several subtypes of ML (Kang and Jameson 2018). Most of the studies used supervised algorithms as depicted in Figure with the most common choice being SVM. However, supervised learning is subject to overtraining and overfitting (Kernbach and Staartjes 2022). Thus, the supervised learning algorithm must therefore be continually retrained to retain a good classification performance when exposed to new input data. Also, while semi-supervised learning can infer new knowledge, supervised learning cannot. The former is of higher importance, given the complexity of AD stages. Due to this, cutting–edge semi-supervised learning such as auto-encoder displayed similar performance to supervised ones such as SVM and CNN. Studies utilizing semi-supervised learning approaches such as stacked auto-encoder (Lao and Zhang 2022) or RNN (Feng et al. 2019) reported accuracy, sensitivities, and specificities over 92%, and 83% for AD vs NC, pMCI versus sMCI binary classification respectively, but utilize limited sample sizes as depicted in Table 2. Consequently, there is a need for research into semi-supervised algorithms for categorizing AD stages.

Although the utilization of metaheuristics with ML has shown promise in various medical domains, including the diagnosis of diseases such as COVID-19, lung cancer, retinal artery veins, chronic kidney, and diabetes, its application in multimodal neuroimaging is relatively limited.

## Comparison with existing studies

There are a few reviews in this research area. Sharma et al. (2023), conducted a multimodal neuroimaging data review that focused on feature selection, feature scaling, and feature fusion. The conclusion for the further study recommended a robust multimodal ML-based classification model trained on features extracted from an in-house created dataset. Nitika and Shamik (Goenka and Tiwari 2022a) focused on brain-imaging biomarkers based on deep learning frameworks. This review to the best of our knowledge gives a detailed overview of research trends in multimodal neuroimaging for AD and analyses them in various strategies namely: fusion level abstraction, ML method, explainability method, and dataset. This survey followed the procedure laid down in Aggarwal et al. (2021) whose focus was basically on the diagnostic accuracy of ML in medical imaging. Table 6 shows the comparison of this survey with existing ones.

**Table 6:** A comparison of our review to the existing survey

Ref	Multimodal data	Analysis based
Sharma and Mandal 2023)	Image	Fusion level of abstraction, feature selection, feature scaling
Goenka and Tiwari 2022a)	Image	neuroanatomy computational, Dataset
Our Study	Image	ML method, Fusion level of abstraction, Explanainbility method, Dataset

## Conclusion

This study shows the potential of multimodal neuroimaging data with machine learning algorithms in accurately classifying different stages of Alzheimer’s Disease. The study performed a systematic review and meta-analysis to evaluate the impact of ML methods on the classification of AD stages. The results show that Machine learning with multimodal neuroimaging data holds great promise for accurately classifying Alzheimer’s disease stages. The study also analyzed the classification algorithms and fusion methods used in the selected studies, providing insights into their strengths and limitations. This information can facilitate researchers in comprehending the diverse methodologies at their disposal and enable them to make judicious choices while devising classification models for Alzheimer’s disease stages, utilizing multimodal neuroimaging. This study also provides the explainability analysis across the selected studies, and it shows that explainability was not available for the majority of the studies, which raises a concern about the reliability of model decisions.

The significant degree of variability or heterogeneity among the research included in the analysis is one of the study’s limitations. This implies that the imaging modalities employed, the image preprocessing methods used, and the classification algorithms used to evaluate the data varied amongst the researchers. Additionally, this review excluded studies that did not report sensitivity and specificity as performance metrics for the classification models. Overall, while the study provides important insights into the potential of machine learning and neuroimaging data for diagnosing AD, these limitations suggest that more research is needed to fully explore and validate these approaches.

Future research should focus on the exploration of other Alzheimer’s disease diagnosis methods with multimodal imaging based on machine learning and metaheuristics approach (Sun et al. 2022; Liu et al. 2021b). The research focus could also be on increasing the sample size for analysis.
